# Morin as a Preservative for Delaying Senescence of Banana

**DOI:** 10.3390/biom8030052

**Published:** 2018-07-12

**Authors:** Hong Zhu, Jiali Yang, Yueming Jiang, Jun Zeng, Xuesong Zhou, Yanglin Hua, Bao Yang

**Affiliations:** 1 Key Laboratory of Plant Resources Conservation and Sustainable Utilization, Guangdong Provincial Key Laboratory of Applied Botany, South China Botanical Garden, Chinese Academy of Sciences, Guangzhou 510650, China; zhuhong@scbg.ac.cn (H.Z.); jlyang@scbg.ac.cn (J.Y.); ymjiang@scbg.ac.cn (Y.J.); junzeng@scbg.ac.cn (J.Z.); 2 University of Chinese Academy of Sciences, Beijing 100039, China; 3 Key Laboratory of Post-Harvest Handling of Fruits, Ministry of Agriculture, Guangzhou 510650, China; 4 Guangzhou Honsea Industry Co., Ltd., Guangzhou 510530, China; zhouxs@honsea.com; 5 Synsun Health Industry Co., Ltd., Guangzhou 510663, China; huayangling@sina.com

**Keywords:** banana, morin, metabolite, nuclear magnetic resonance spectroscopy (NMR), senescence

## Abstract

Banana is a climacteric fruit with desirable palatability and high nutritional value. It ripens rapidly accompanied with metabolite changes during postharvest storage. In this work, morin was applied to treat banana to delay senescence. Nuclear magnetic resonance (NMR) spectroscopy was used to monitor the changes of metabolite composition and levels in banana. The results showed that morin significantly delayed the changes of color and firmness. 1D and 2D NMR spectra reflected that the levels and composition of metabolites were changed with the senescence initiation. The principal component analysis revealed that the first principal components responsible for banana senescence were carbohydrates, amino acids, lipids and phenolics. Morin treatment delayed the transformation of starch to glucose, fructose and sucrose, accelerated the accumulations of alanine and γ-Amino-butyrate (GABA), postponed the generations of valine and l-aspartic acid, suppressed the degradation of saponin a. It indicated that morin was effective in delaying banana senescence.

## 1. Introduction

Banana fruit is an important horticultural crop with high nutritional value. It is rich in soluble sugars, vitamins, carotenoids, phenolics and minerals [1,2]. Various bioactivities and therapeutic properties have been identified for banana. Unripe banana extract exhibits antiulcer activity against aspirin-induced ulceration through stimulating the growth of gastric mucosa [3]. Owing to its antimicrobial activity, banana can be used for prophylactic treatment in preventing cardiovascular dysfunction and muscular degeneration [4]. The antioxidant capacity has been well documented for bioactive compounds in banana, including carotenoids, phenolics and phytosterols [5]. Moreover, consumption of banana can reduce the incidence of cancer [6].

Physicochemical and biochemical attributes of banana change during senescence. The surface color changes from green to yellow, and the texture softens. Starch degrades into sucrose and glucose leading to sweet taste. These changes contribute to banana with good appearance and desirable quality. However, banana is a typical climacteric fruit and deteriorates rapidly during the late phase of senescence. Therefore, an efficient preservation technique is required to maintain the quality and extend the shelf life of banana [7]. A variety of physical and chemical approaches have been tested to inhibit the growth of pathogens and to delay the physiological senescence [1,8,9]. Chemical treatment has been considered as an efficient technique [10]. Morin is a multiple-hydroxyl flavonoid present in Moraceae plants. Due to its impressive biological activities, morin has been developed as a chemopreventive agent against oral carcinogenesis, a potential anti-angiogenic drug via enhancing the endostatin level, and an antioxidant to protect cells from hydrogen peroxide-induced damage [11]. It has also been reported as an antimicrobial agent against *Streptococcus mutans* and other pathogens [12]. Therefore, it is interesting to investigate the preservative effect of morin on banana.

Nuclear magnetic resonance (NMR) spectroscopy is a promising tool to identify and quantify complex metabolites from postharvest crops [13,14]. Principal component analysis (PCA) has been considered as a common and powerful multivariate technique in metabolomics studies and provides insights into the separation between experimental groups based on data of mass spectrometry, NMR or other analytical techniques [15]. The combination of NMR and PCA allows the analysis of metabolites profiles and the identification of featured metabolites responsible for the significant differences due to treatment.

The current study aimed to investigate the preservative effects of morin on banana during postharvest storage. The color and firmness were measured to evaluate the ripeness of banana. The metabolites profiles at different ripening stages were determined by NMR spectroscopy. The principal metabolites responsible for the delayed senescence of morin-treated banana by analysed by PCA.

## 2. Material and Methods

### 2.1. Plant Materials and Reagents

Fruits of banana (Musa AAA Giant Cavendish cv. Baxi) at green mature stage were collected from a local market in Guangzhou, China. Methanol-*d*_4_ (CD_3_OD) was obtained from Energy Chemical (Shanghai, China). Morin was bought from Macklin Biochemical (Shanghai, China). Other reagents were of analytical grade.

### 2.2. Fruit Treatment

The fruit sample of uniform size, shape and maturity were selected and divided into two groups randomly. Morin was dissolved in ethanol and further diluted by water to a final concentration of 0.1% (*w*/*w*) was applied to immerse bananas for three minutes. All fruits were stored at 25 °C. The skin color and firmness were tested after storing for 0, 10, 20 and 30 days. The metabolites profiles were analyzed by NMR.

### 2.3. Measurement of Skin Color

Skin color of banana sample was measured by a Minolta Chroma Meter CR-400 (Minolta Camera Co. Ltd., Osaka, Japan). Three fruit fingers were withdrawn randomly from each stage. Three equidistant points around the middle position of the surface were picked and used for the measurement of skin color. Three color scales of L*, a* and b* were recorded. L indicates the lightness or darkness, a* represents green to red color and b^*^ denotes blue to yellow color. These three numerical parameters were converted into the hue angle (h^0^) according to the following formula:

h^0^ = tan^−1^ (b*/a*)

### 2.4. Determination of Fruit Firmness

Fruit firmness was measured by a penetrometer (ModelGY-3, Zhejiang Scientific Instruments, Zhejiang, China) with a probe of 0.6 cm in diameter. Three fruit fingers were withdrawn randomly from each stage. Each finger was measured at three equidistant points around the middle position of the fruit. The force required to penetrate into the fruit was recorded. The final results were expressed as the ratio of the force to the applied force area (kg/cm^2^).

### 2.5. Analysis by NMR Spectroscopy

The fruit peel was frozen after liquid nitrogen treatment and was crushed into powder. 250 mg of sample were weighed and mixed with 1 mL of Methanol-*d*_4_. Ultrasound treatment was applied to extract metabolites from banana peel. The extraction was carried out at 40 °C in a water bath for 30 min. The extract was centrifuged at 5000 *g* for 10 min, and the supernatant was collected and stored in a 5-mm magnetic tube (ST500-7, Norell, Morganton, NC, USA) for NMR analysis. All assays were conducted in three replicates. Morin-treated group at four stages were sampled and named as Morin-0-1, Morin-0-2, Morin-0-3, Morin-10-1, Morin-10-2, Morin-10-3, Morin-20-1, Morin-20-2, Morin-20-3, Morin-30-1, Morin-30-2, Morin-30-3. Control group at four stages were sampled and named as CK-0-1, CK-0-2, CK-0-3, CK-10-1, CK-10-2, CK-10-3, CK-20-1, CK-20-2, CK-20-3, CK-30-1, CK-30-2 and CK-30-3.

^1^H NMR spectra were acquired from a BRUKER AVANCE III 500 spectrometer (Bruker, Karlsruhe, Germany) at 500.13 MHz, equipped with *Z*-gradient system at 25 °C. Standard 2D NMR analyses were also performed for signal assignment including ^1^H-^1^H correlation spectroscopy (COSY), selective total correlation spectroscopy (1D-TOCSY), J-resolved spectroscopy (JRES), ^1^H-^13^C heteronuclear single quantum coherence spectroscopy (HSQC) and heteronuclear multiple bond correlation (HMBC). For COSY and 1D-TOCSY assays, 128 transients were collected into 1024 data points for each of 160 increments. Phase insensitive mode with gradient selection was employed in COSY assays. MLEV-17 functioned as the spin-lock scheme in the phase sensitive 1D-TOCSY assay with mixing time of 80 ms. A spectral width of 12.0 ppm was applied for every dimension. For JRES spectra, 128 transients were collected into 4096 data points for each of 80 increments. The spectral width was set as 6000 and 60 Hz for the acquisition and evolution dimension, respectively. For ^1^H-^13^C HSQC and HMBC assays, spectra were recorded by using the gradient selected sequences with 512 transients and 2048 data points for each of 128 increments. The spectral width was set as 6000, 27,500 and 20,625 Hz for ^13^C HSQC assays and ^1^H, ^13^C HMBC assays, respectively. All data were zero-filled to a 2048 × 2048 matrix with appropriate window functions prior to Fourier transformation.

### 2.6. Processing and Analysis of NMR Data

TOPSPIN (v3.2, Bruker Biospin, Germany) was employed to analyze ^1^H NMR spectra of banana peel extract. The chemical shift 3.31 ppm of MeOD-*d*_4_ functioned as a reference to calibrate phase and baseline distortions. Metabolites in methanol extract were determined according to proton and carbon signals in 1D and 2D NMR spectra. Their relative contents were calculated based on the area of characteristic peaks in ^1^H spectra, where a linear baseline scaling normalization approach was adopted. The baseline was constructed by calculating the median of each feature over all spectra. The scaling factor was measured for each spectrum as the mean intensity ratio of the baseline to the spectrum. The intensity of each spectra was multiplied by its particular scaling factor [16]. SIMCA 15 (Umetrics, Malmo, Sweden) was applied to analyze the principal components and to find the general trend and possible outlier. R^2^X and Q^2^ values were computed to reflect the explained variables and prediction ability of the model.

### 2.7. Statistical Analysis

All experiments were arranged in completely randomized design. Triplicate assays were performed for the measurement of all parameters. The results were expressed as mean ± standard error and subjected to variance analysis. The least significant difference was set as *p* < 0.05.

## 3. Results and Discussion

### 3.1. Changes of Hue Angle and Firmness

**Figure 1:**
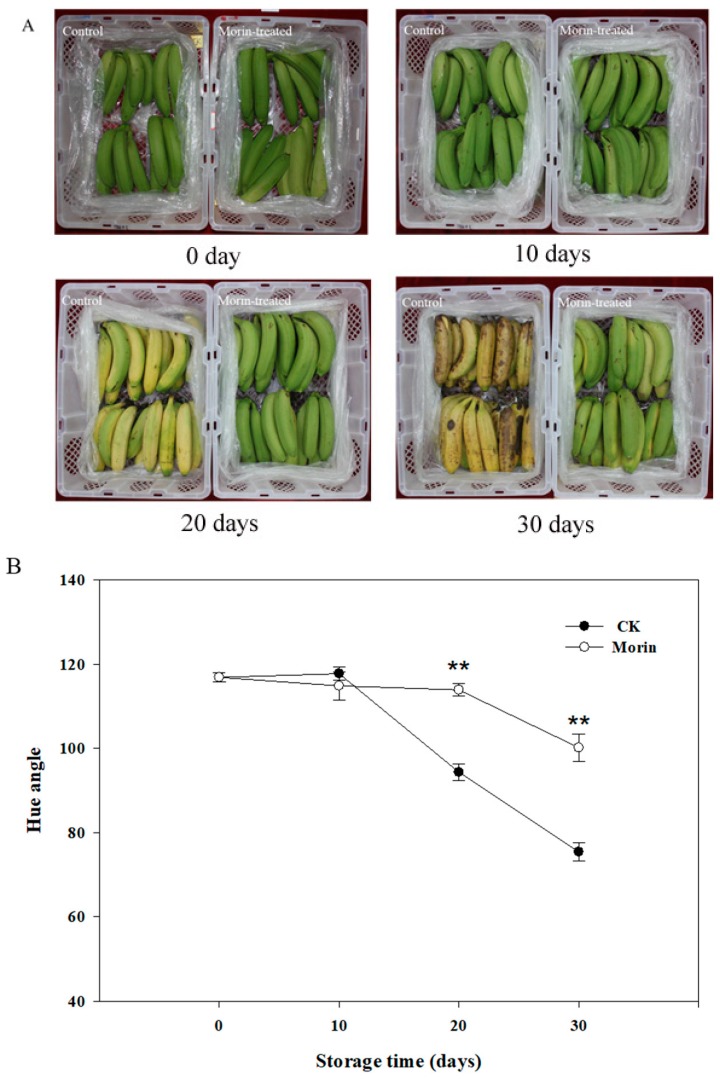

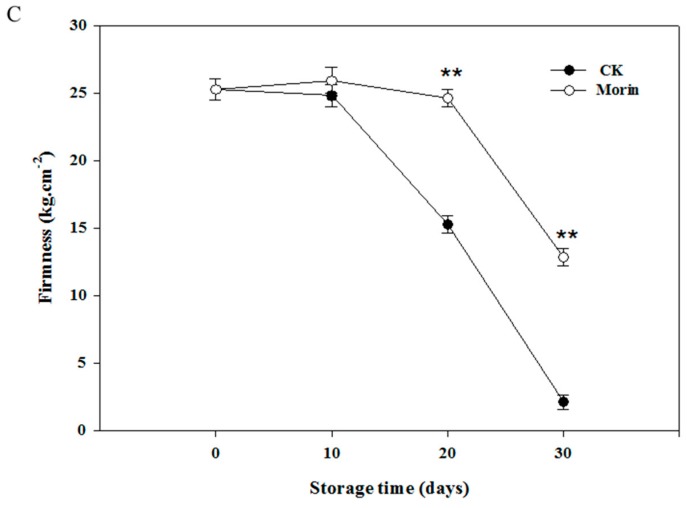
The changes of appearance (**A**); color (**B**); and firmness (**C**) of banana during storage. For (**B**,**C**): •, CK, control; ○, 0.1% morin-treated. Data were presented as the means ± standard errors. Asterisks represent the significant difference between the control and morin-treated groups at the same time (** *p* < 0.01).

The appearances of banana fruit at four postharvest stages are shown in Figure 1A. When storing for 10 to 20 days, the skin color of banana fruit in the control group changed from green to yellow. After 30 days storage, brown spots appeared severely accompanied with severe fungal infections, but in the morin-treated group, the skin color started to change to yellow after 30 days storage, neither brown spot nor fungal infection was found on the skin. Consistent with the appearance, the hue angle value changed dramatically from 10 to 30 days in the control group, while this value changed much slower in the morin-treated group and maintained at a high level after 30 days storage (Figure 1B). The changing skin color was associated with the breakdown of chlorophyll. The appearance of spots indicated that banana was in the deterioration stage. Therefore, the skin color and spots are important markers defining the quality and senescence of banana fruit. Firmness is another physical parameter to assess the extent of senescence. As shown in Figure 1C, banana in the control group began to soften rapidly after 10 days storage and became unacceptable after 30 days, while the fruit maintained a high firmness (12.85 ± 0.64 kg·cm^−2^) after being treated by 0.1% morin. Therefore, morin was effective to maintain banana quality and to delay the senescence of banana fruit and to inhibit fungal infection during postharvest storage.

### 3.2. Identification of Metabolites in Banana Peel Extract by NMR

**Figure 2:**
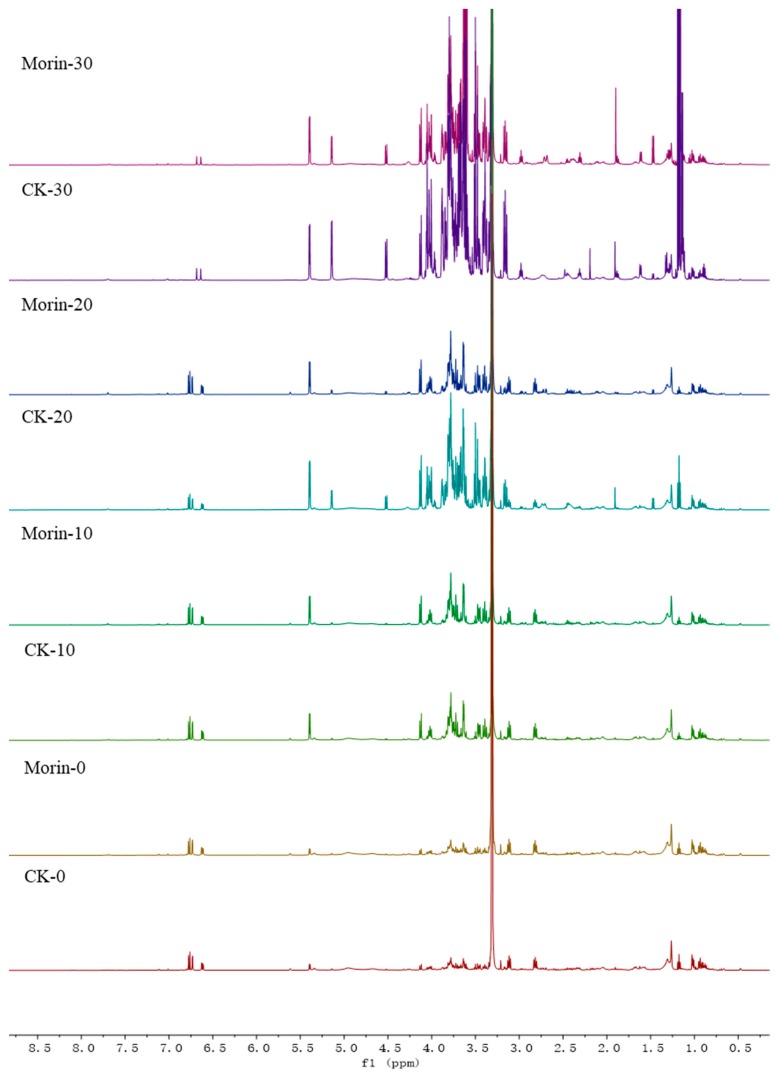
^1^H NMR spectra of banana peel extracts from four stages. From bottom up, CK-0; Morin-0; CK-10; Morin-10; CK-20; Morin-20; CK-30; Morin-30.

**Figure 3:**
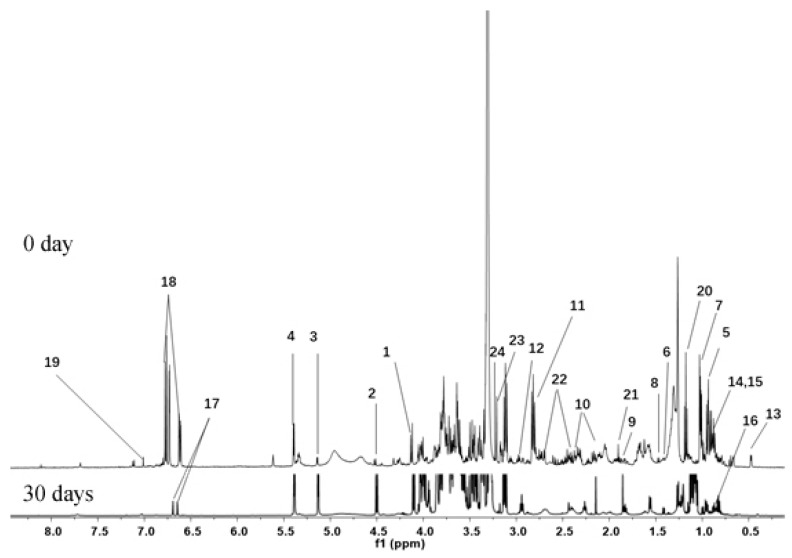
Representative ^1^H NMR spectra of banana peel extracts. The lower spectra is from banana with 30 days storage; the upper spectra is from banana with 0 days storage. Peaks: 1, fructose; 2, β-d-glucose; 3, α-d-glucose; 4, sucrose; 5, leucine; 6, valine; 7, isoleucine; 8, alanine; 9, γ-amino butyrate; 10, glutamine; 11, l-aspartic acid; 12, asparagine; 13, saponin a; 14, palmitic acid; 15, linoleic acid; 16, saponin b; 17, salsolinol; 18, dopamine; 19, gallic acid; 20, ethanol; 21, acetic acid; 22, malic acid; 23, choline; 24, phosphocholine.

In this work, the metabolite profiles of banana fruit with or without morin treatment at four stages were analyzed by 1D and 2D NMR. The identified metabolites and their chemical shifts are presented in Table 1. As shown in Figure 2, ^1^H NMR spectra of banana fruit with or without morin treatment were compared at four stages. The quantitative and qualitative differences were observed. Twenty-four compounds were identified. Their representative proton signals are marked in Figure 3. Most of them were primary metabolites and were involved in plant growth and development directly. α-d-glucose, β-d-glucose, sucrose, fructose were the leading carbohydrates identified, while some free amino acids were observed, including alanine, asparagine, γ-aminobutyric acid, glutamine, isoleucine, l-aspartic acid, leucine and valine. Palmitic acid and linoleic acid were the lipids in banana peel. Three phenolics, dopamine, salsolinol and gallic acid were identified from specific ripening stages. Other small molecules were characterized as acetic acid, choline, ethanol, malic acid and phosphocholine.

**Figure 4:**
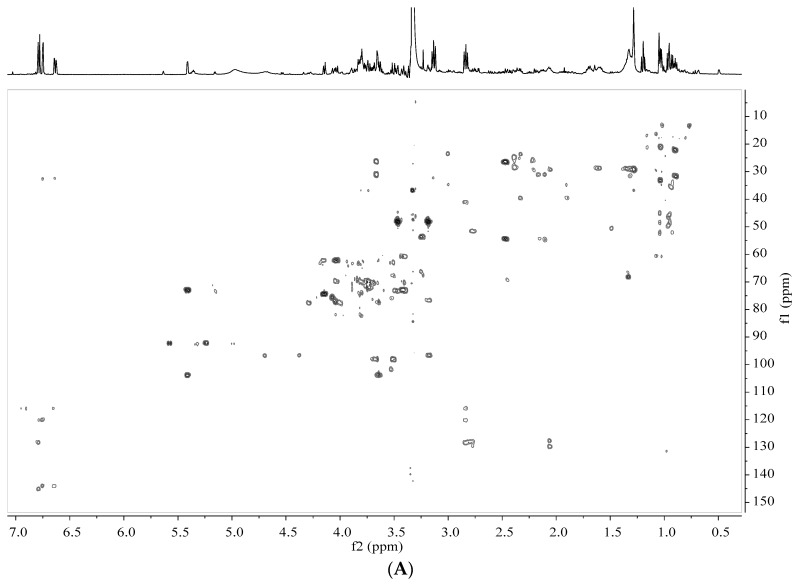

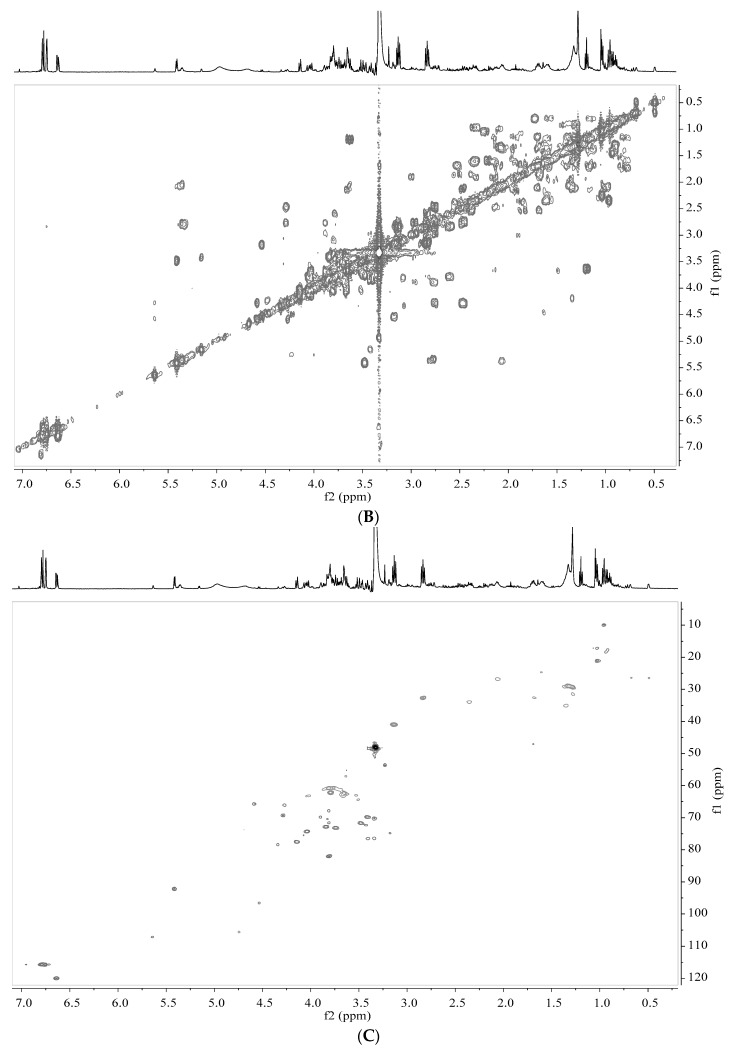

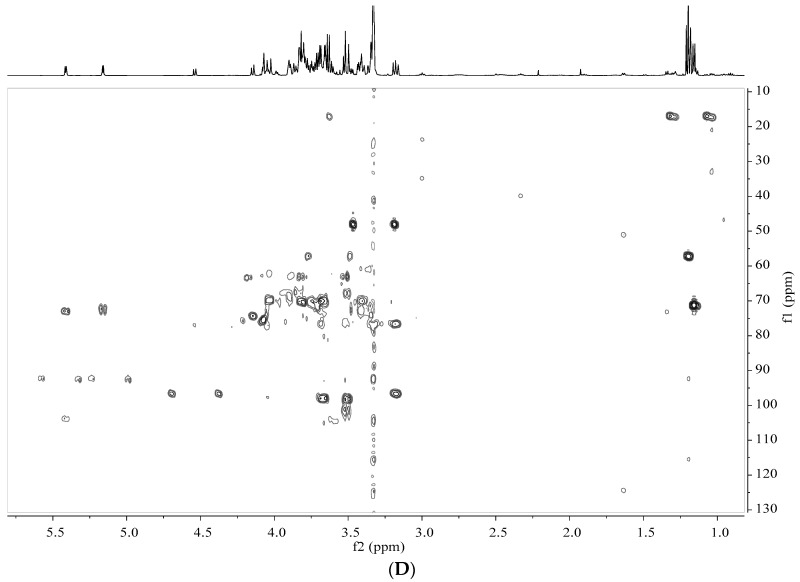
NMR spectra of banana peel extracts. (**A**) Heteronuclear multiple bond correlation (HMBC) spectra at 0 days of control; (**B**) Correlation spectroscopy (COSY) spectra at 0 days of control; (**C**) Heteronuclear single quantum coherence spectroscopy (HSQC) spectra at 0 days of control; (**D**) HMBC spectra at 30 days of control.

In the ^1^H NMR spectra, overlapped signals from 3.0 to 5.5 ppm were assigned to carbohydrates. Their major differences were characterized by their anomeric signals. An anomeric proton signal at 4.13 ppm (d, *J* = 8.6 Hz) was assigned to fructose, signal at 4.52 ppm (d, *J* = 8.0 Hz) was β-d-glucose, signal at 5.14 ppm (d, *J* = 3.72 Hz) was α-d-glucose, signals at 5.40 ppm (d, *J* = 3.8 Hz) was sucrose [17]. Other proton signals of these sugars were assigned at the range of 3.0–4.0 ppm in NMR spectra. Data of COSY, HSQC and HMBC spectra are shown in Figure 4A–D.

The multiplets at 1.89 and triplets at 2.31 (*J* = 7.5 Hz), 2.98 ppm (*J* = 7.5 Hz) were assigned to three methylene groups of γ-aminobutyrate (GABA). Other free amino acids were identified by our previous work [13]. Chemicals derived from lipid metabolism were also characterized. The proton signals at 0.89 (s), 1.20–1.33 (m), 1.54–1.64 (m), 2.24 (s) and 2.38 (s) were assigned to palmitic acid and linoleic acid. The proton signals at 0.48 (d, *J* = 3.9 Hz) and 0.66 ppm (d, *J* = 4.0 Hz) were assigned to saponin a. The single peek at 0.89, double peaks at 1.62 (*J* = 6.7 Hz), multiplets at 1.30–1.33 ppm were assigned to saponin b.

Signals in the range of 6.5–8.0 ppm from ^1^H NMR spectra were assigned to phenolics [18]. Three aromatic protons at 6.78 (d, *J* = 8.0 Hz), 6.73 (d, *J* = 2.3 Hz), 6.62 ppm (d, *J* = 8.0, 2.3 Hz) indicated the existence of an important secondary metabolite. Further analysis of ^1^H-^1^H COSY spectra led to the identification of benzene ring with ABX spin system. In the HSQC experiments, H-5 (6.77 ppm) correlated with C-5 (115.5 ppm), H-2 (6.73 ppm) correlated with C-2 (115.8 ppm) and H-6 (6.62 ppm) correlated with C-6 (120.1 ppm). In the HMBC experiments, the ^3^*J* long-range correlations from H-2 and H-6 to C-7 (32.1 ppm) offered additional evidences. Thus, these three protons were assigned to dopamine. Surprisingly, these three protons disappeared completely and two aromatic protons at 6.68 (s) and 6.64 ppm (s) appeared subsequently in ^1^H NMR spectra after 30 days storage. Further analyses of HSQC confirmed the correlations from H-2 (6.66 ppm) to C-2 (114.5 ppm) and from H-5 (6.70 ppm) to C-5 (112.0 ppm). In the HMBC spectra, the long-range correlations from H-2 to C-7 (24.3 ppm), C-4 (144.2 ppm), C-6 (124.6 ppm) and from H-5 to C-9 (51.5 ppm), C-3 (145.1 ppm), C-1 (122.4 ppm) were observed, which confirmed the transformation of dopamine to salsolinol [19].

The peeks at 1.18 (t, *J* = 7.5 Hz) and 3.62 ppm (m) were identified as the signals of ethanol. Other single peaks were also assigned. The signal at 1.91 ppm was assigned to acetic acid, while 3.21 ppm was choline and 3.23 was phosphocholine. Other metabolites, e.g., malic acid and gallic acid, were also identified from banana peel extract.

### 3.3. Principal Component Analysis

**Figure 5:**
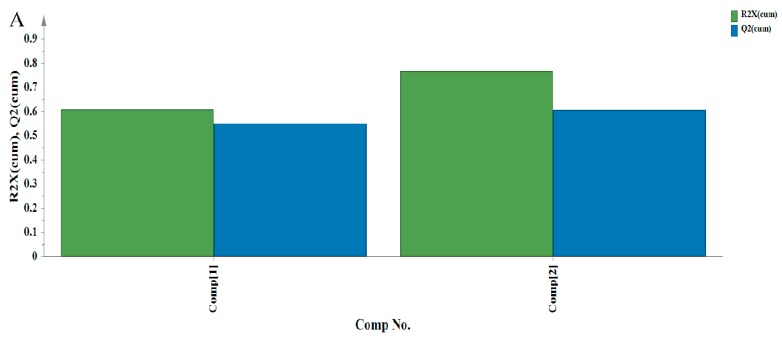

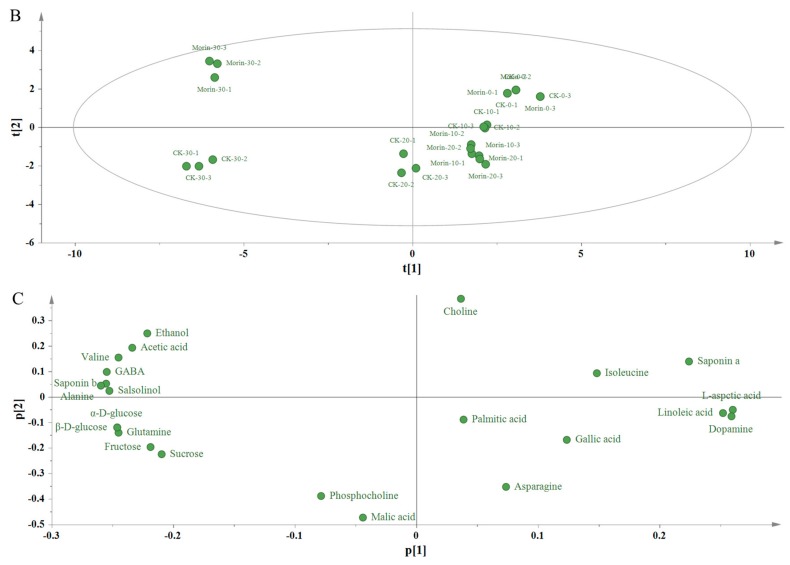
Principal component analysis (PCA) results for the metabolites profile of banana peel extracts. (**A**) R^2^X and Q^2^ graph; (**B**) the score plot of PCA; (**C**) the loading plot of PCA.

The qualitative analyses of metabolites in banana peel extract were conducted based on the characteristic peaks in the NMR spectra, while the quantitative analysis was performed according to the area of each peak. The effect of morin treatment on the metabolites changes in banana peel extracts during the ripening process was analysed by PCA. An unsupervised approach was applied for PCA in this study. As shown in Figure 5A, 76.8% of the total variance could be explained by the first two principal components, in which 61% could be explained by the first principal component. The value of R^2^X was 0.993 which indicated a good fitness of the model.

The score plot was employed to analyze the statistical similarity among all samples. Three replicates were collected from the morin-treated group and the control group in each stage. As shown in Figure 5B, the triplicates of banana samples were clustered tightly, indicating that the experimental protocol possessed a good repeatability. Morin-treated banana and untreated banana at 0 day clustered together in the first quadrant. No significant metabolite differences were found for 10 and 20 days storage for the morin-treated group as they clustered closely in the fourth quadrant. Control groups at 10 days, 20 days, 30 days storage and the morin-treated group at 30 days storage were isolated from each other and located in the positive part of horizontal axis, the third quadrant and the fourth quadrant, respectively. These results indicated that there was a significant change in the metabolomic profiles. Significant differences between the morin-treated group and control group were observed for 10 days to 30 days storage.

The loading plot was applied to analyze the principal components responsible for the quality changes between the morin-treated group and the control group during senescence. As shown in Figure 5C, the first principal components were mainly characterized by acetic acid, α-d-glucose, alanine, β-d-glucose, dopamine, ethanol, fructose, GABA, glutamine, isoleucine, l-aspartic acid, linoleic acid, salsolinol, saponin a, saponin b, sucrose, and the second principal components were represented by choline, gallic acid, malic acid and phosphocholine.

Carbohydrates are the principal components in banana fruits. Their metabolisms are tightly associated with the quality change during postharvest ripening [20]. Their composition and contents were related to the sweetness and texture of banana. The content of sucrose increased remarkably during storage. For the control group, its content reached the peak at 20 days storage and was maintained at a high level at the last 10 days storage. While for the morin-treated group, its content kept increasing slightly during the four stages. Fructose is the enzymatic hydrolysate of sucrose and exhibited a similar change trend of sucrose. The accumulations of α-d-glucose and β-d-glucose changed little (*p* = 0.54, *p* = 0.18) in the early 10 days but increased dramatically (*p* = 0.009, *p* = 0.008) in the later 20 days. Its growth rate in the morin-treated group was much slower than that in the control group as the final concentration of morin-treated banana was half of untreated banana. Starch is the major carbohydrate in green banana, but its content drops rapidly during climacteric periods accompanied with the accumulation of its hydrolysates, reducing sugars and sucrose [21]. Therefore, morin treatment might delay the occurrence of respiration climacteric leading to the delayed transformation of starch to glucose.

Amino acids are important nutrients in banana, and their metabolisms are dramatic during the postharvest storage. Alanine and glutamine were the two leading amino acids responsible for the metabolite changes. They increased remarkably from 0 to 30 days storage. The increasing rate of alanine was lower in the first 20 days but much higher in the last 10 d after morin treatment. While for glutamine, its increasing rate declined after morin treatment. The content of GABA changed insignificantly from 0 to 10 days storage but increased significantly after 10 days storage. At 30 days storage, the content of GABA in the morin-treated group was obviously higher than that in the control group. Since glutamine can turn into glutamate under the action of enzymes. GABA might be transformed from glutamate via glutamate decarboxylase. Therefore, the biosynthesis of GABA was in accordance with the ‘GABA shunt’ pathway [22]. The glutamate–glutamine cycle is an important neurotransmitter cycling metabolism process to supply adequate neurotransmitter glutamate for the central nervous system, where glutamine is converted to glutamate and then metabolized into GABA. Alanine plays an important role in ammonia homeostasis [23]. Thus, morin might stimulate the accumulation of alanine and GABA, which led to a higher nutritional value for banana.

Isoleucine, l-aspartic acid and valine were the other principal amino acids at four stages. The content of isoleucine decreased during storage. No significant differences were observed between the two groups. For l-aspartic acid, its content in the control group changed insignificantly from 0 to 10 days storage but decreased rapidly in the following storage, while in the morin-treated group, it decreased from 20 days storage onwards. For valine, the change trend was similar to that of l-aspartic acid. Therefore, amino acids in banana changed during storage, and the slower changes led to delayed senescence.

Lipid metabolism, especially membrane lipids metabolism, is responsible for plant senescence [24]. Linoleic acid and palmitic acid were the two representative fatty acids identified from NMR spectra. Linoleic acid is the lipid in cell membranes and degraded during storage which might be associated with the ripening process. No significant changes were found for palmitic acid. Saponins belong to a class of lipophilic triterpenoid. They can protect the plants against microbes and fungi [25]. Saponin a and saponin b were the principal saponins in banana and contributed to the quality differences during storage. Saponin a decreased rapidly after 20 days storage in the control group, while its decreasing rate was much slower after morin treatment. Saponin b was observed at 30 days storage, and its content in the control group was higher than that in the morin-treated group. The results were in consistence with the appearance of banana during storage. As spots appeared only in the control group at 30 days storage, the low content of saponin a might be associated with the weak anti-fungi activity in banana.

Dopamine is an important phenolic compound in banana peel extract and exhibits strong antioxidant activity [26]. It can serve as a neurotransmitter in brain and can produce the feeling of pleasure [27,28]. The content of dopamine in the control group decreased after 10 days storage and no dopamine could be detected in 30 days storage. In contrast, salsolinol could not be detected in the former 20 days, but only in 30 days storage. Salsolinol might be the reaction product of dopamine and acetaldehyde through Pictet–Spengler condensation [29]. Though these three metabolites shared the same variation tendency in both control group and morin-treated group, the accumulation of salsolinol was delayed after treated by morin, while accumulation of dopamine was accelerated in the former 20 days.

Acetic acid, choline, gallic acid, malic acid and phosphocholine were the common primary metabolites in banana. These compounds belonged to the first two principal components in the loading plot, and might be associated with carbohydrate metabolism, protein metabolism and lipid metabolism mediated by a sequence of endogenous enzymes during senescence.

## 4. Conclusions

In conclusion, morin delayed the skin color change from green to yellow, slowed down senescence, and inhibited fungal infection. NMR was a useful tool to characterize the metabolites in banana. Twenty-four chemicals were identified, and their contents were determined. PCA analysis indicated that a significant effect of morin on metabolite changes occurred after 10 days storage, including the delayed accumulation of sucrose, fructose, α-d-glucose and β-d-glucose, the accelerated accumulation of alanine and GABA, the retarded formation of valine and l-aspartic acid, the suppressed degradation of saponin a. It suggested that morin was a promising preservative for banana to maintain the quality and extend shelf life.

## Figures and Tables

**Table 1:** Assignment of proton signals in ^1^H nuclear magnetic resonnance (NMR) spectra (MeOD-*d*_4_).

No.	Metabolites	Assignment of Proton Signals
	**Carbohydrates**	
1	Fructose	4.13 (d, *J* = 8.6 Hz)
2	β-d-glucose	4.52 (d, *J* = 8.0 Hz), 3.16 (m)
3	α-d-glucose	5.14 (d, *J* = 3.72 Hz)
4	Sucrose	5.40 (d, *J* = 3.8 Hz), 3.84 (m), 3.74 (m), 3.47 (m), 3.41 (m)
	**Amino acids**	
5	Leucine	0.92 (d, *J* = 7.0 Hz), 0.94 (d, *J* = 7.0 Hz)
6	Valine	1.00 (d, *J* = 7.0 Hz), 1.05 (d, *J* = 7.0 Hz)
7	Isoleucine	1.02 (d, *J* = 7.0 Hz), 0.95 (t, *J* = 7.5 Hz)
8	Alanine	1.47 (d, *J* = 7.2 Hz), 3.64 (m)
9	γ-Amino-butyrate (GABA)	1.89 (m), 2.31 (t, *J* = 7.5 Hz), 2.98 (t, *J* = 7.5 Hz)
10	Glutamine	2.12 (m), 2.47 (m), 3.66 (m)
11	l-aspartic acid	2.57 (m), 2.80 (m), 3.78 (m)
12	Asparagine	2.73 (m), 2.93 (m), 3.87 (m)
	**Lipids**	
13	Saponin a	0.48 (d, *J* = 3.9 Hz), 0.66 (d, *J* = 4.0 Hz)
14	Palmitic acid	0.89 (t, *J* = 7.3 Hz), 1.20–1.28 (m), 1.54–1.58 (m), 2.24 (t, *J* = 7.5 Hz)
15	Linoleic acid	0.89 (t, *J* = 7.3 Hz), 1.30–1.33 (m), 1.58–1.64 (m), 2.38 (t, *J* = 7.5 Hz)
16	Saponin b	0.89 (s), 1.30–1.33 (m), 1.62 (d, *J* = 6.7 Hz)
	**Phenolics**	
17	Salsolinol	6.68 (s), 6.64 (s)
18	Dopamine	6.78 (d, *J* = 8.0 Hz), 6.73 (d, *J* = 2.3 Hz), 6.62 (d, *J* = 8.0, 2.3 Hz)
19	Gallic acid	7.01 (s)
	**Other metabolites**	
20	Ethanol	1.18 (t, *J* = 7.5 Hz), 3.62 (m)
21	Acetic acid	1.91 (s)
22	Malic acid	2.45 (m), 2.75 (m), 4.27 (m)
23	Choline	3.21 (s)
24	Phosphocholine	3.23 (s)
